# The roles of biomarkers in Alzheimer's disease clinical trials

**DOI:** 10.1016/j.neurot.2025.e00811

**Published:** 2025-12-24

**Authors:** Jeffrey Cummings, Shailja Sharma, G. DeAndrea, Amanda Leisgang Osse, Andrew Ortiz

**Affiliations:** a Chambers-Grundy Center for Transformative Neuroscience, Department of Brain Health, Kirk Kerkorian School of Medicine, University of Nevada Las Vegas (UNLV), Las Vegas, NV, USA; b Department of Brain Health, Kirk Kerkorian School of Medicine, University of Nevada Las Vegas (UNLV), Las Vegas, NV, USA; c Kirk Kerkorian School of Medicine, University of Nevada Las Vegas (UNLV), Las Vegas, NV, USA

**Keywords:** Biomarker, Alzheimer's disease, Clinical trials, Context of use, Amyloid imaging, p-Tau 217

## Abstract

Biomarkers are essential to guide decision making in Alzheimer's disease (AD) clinical trials where they have a variety of contexts of use (COUs) including diagnosis, risk, pharmacodynamic response, prognosis, prediction, monitoring, and safety. The COU of biomarkers may differ by phase of drug development with Phase 1, 2, and 3 emphasizing different types of information for decision making. A variety of biomarkers are currently serving as pharmacodynamic outcomes in clinical trials including amyloid and tau PET and fluid measures of amyloid, tau, neurodegeneration, inflammation, and synaptic plasticity. Biomarker strategies are integrated throughout drug development programs from collection and assay performance to statistical analysis and data interpretation. Data interrogation approaches using artificial intelligence and machine learning may enhance the value of biomarker observations through integration of multimodal data. Emerging biomarkers that may play a role in future AD trials include proteomics, exosome assays of co-pathology occurring in AD, EEG, ocular measures, and digital biomarkers. Biomarkers inform drug development decision-making including termination of candidate agents without sufficient biomarker effects, resourcing of promising therapies impacting the fundamental features of AD, and accelerating the development of new therapies for those with or at risk for AD.

Alzheimer's disease (AD) diagnostics and therapeutics are making remarkable progress. After a hiatus of nearly 20 years with no approvals of new classes of agents, three anti-amyloid monoclonal antibodies (MABs) have been approved [1]. Donanemab and lecanemab are available on the market in the US and in several other countries [2]. Disease targeted therapies (DTTs) aimed at ameliorating tau pathology, clearing amyloid species, reducing inflammation, improving metabolic and bioenergetic function, enhancing synaptic plasticity, and addressing other targets are active in the AD drug development pipeline [3]. Success in developing new treatments for AD builds on a growing foundation of understanding of biomarkers for AD. Imaging, cerebrospinal fluid (CSF), and blood-based biomarkers are increasingly accessible to help guide decision making in AD clinical trials [4]. Biomarkers are now indispensable for DTT development.

We review the definition of biomarkers, describe the contexts of use (COU) of biomarkers in AD clinical trials, discuss the use of biomarkers in each phase of drug development, and address special issues in the use of biomarkers in AD trials settings including combination trials and platform trials. We describe biomarkers serving as pharmacodynamic outcomes to assess efficacy in AD trials. We note emerging biomarkers that may play a role in future trials, and we discuss analytic approaches that assist in interpreting biomarker results from trials. We provide examples from recent trials to illustrate the use of biomarkers in AD clinical trials [5]). Standards for the minimal acceptable performance of blood-based biomarkers have been published and are not described here [6].

## Definition and Context of Use of Biomarkers

A biomarker is defined as characteristics that can be measured and is an indicator of abnormal biological processes, pathogenic processes, or responses to an exposure or intervention [7,8]. Reporting of a biomarker depends on three foundational components: the materials required for performing the measurement, an assay for obtaining the measurement of the biomarker of interest, and a method or criteria for interpreting the measurement. The usefulness of the biomarker will depend on the scientific rigor with which these three elements are established. Biomarker measures can be affected by some systemic conditions, medications, or the ethnoracial background of the individual. These possible confounding factors must be accounted for when determining if an abnormal test is attributable to AD pathophysiology [[9], [10], [11]]. Biomarkers are distinguished from clinical outcome assessments that measure how an individual feels, functions, or survives. Correlations between biomarker outcomes and clinical outcomes are key to interpreting the significance of biomarker changes in response to an intervention.

COU defines how a biomarker will be used in a clinical trial (Table 1). Diagnostic biomarkers confirm or establish the patient's diagnosis; susceptibility/risk biomarkers identify individuals who have an increased or decreased chance of developing a disease or condition; prognostic biomarkers forecast the likelihood of disease progression or recurrence; pharmacodynamic (response) biomarkers measure the biological response related to a therapy; monitoring biomarkers detect the change in disease when measured sequentially; predictive biomarkers indicate the likely benefit or harm in response to the treatment; and safety biomarkers identify and monitor adverse events of a therapy [7,8]. Table 1 provides examples of biomarkers that have served varying COUs in AD clinical trials.

**Table 1:** Biomarkers used in Alzheimer's disease clinical trials according to their context of use.

Context of Use	Definition	Examples of Biomarker Context of Use in Alzheimer's Disease Clinical Trials
Diagnosis	A biomarker that is used to detect or confirm a medical condition or classify individuals based on the subtype of the disease	• Amyloid PET • Tau PET • Pasma p-tau 217 • CSF hybrid ratios p-tau181/A*β*42, t-tau/A*β*42, A*β*42/40
Susceptibility/risk	A biomarker that identifies the risk an individual has of developing a medical condition in the absence of current clinical signs or symptoms	• APOE4 allele • Autosomal dominant AD mutation • Diabetes • Obesity
Prognosis	A biomarker that provides prognostic events such as recurrence and progression in individuals already diagnosed with a medical condition	• Level of plasma p-tau 181 • Plasma p-tau 217 • FDG PET
Pharmacodynamic (response)	Target engagement: agent reacts with intended target	• Pharmacologic response directly related to the mechanism of action of the drug • Appropriate biomarkers vary for each candidate therapy
	Disease impact: Indicates that agent produces effect on the disease pathology	• Amyloid PET • Tau PET • CSF total tau and p-tau • Plasma p-tau 181, p-tau 217, GFAP
	Surrogate biomarker: a Biomarker that serves as replacement for clinical endpoint with the expectation that it predicts benefit or harm from an intervention	• There are no fully validated surrogate markers for AD • Reduction in amyloid plaque on amyloid PET is acceptable for accelerated approval for treatment of individuals with symptoms consistent with AD based on the reasonable likelihood of the relationship between amyloid plaque lowering and clinical benefit
Monitoring	A biomarker used longitudinally to assess disease progression or treatment response	• Plasma p-tau 181, p-tau 217, GFAP • CSF p-tau181/A*β*42, t-tau/A*β*42, A*β*42/40
Predictive	A biomarker used to distinguish susceptibility to negative or positive treatment effects at the time of intervention	• APOE4 allele (predicts increases likelihood of ARIA) • Tau PET may predict patients less likely to respond to anti-amyloid MABs
Safety	A biomarker used to evaluate likelihood and extent of toxicity from exposure to a therapeutic treatment	• MRI detection of ARIA • ECG • Liver function tests • Other laboratory assessments

AD – Alzheimer's disease; APOE4 – apolipoprotein E ε4; ARIA - amyloid related imaging abnormalities; CSF - cerebrospinal fluid; ECG - electrocardiogram; FDG - fluorodeoxyglucose; GFAP - glial fibrillary acidic protein; MABs – monoclonal antibodies; MRI - magnetic resonance imaging; PET - positron emission tomography.

Biomarkers include various types of brain imaging and biofluid studies (e.g., analytes in blood, CSF, etc). Imaging studies provide detailed information on the location and intensity of pathology and reflect the changes that have occurred cumulatively over time (e.g., atrophy, or amyloid of tau deposition). Imaging is often costly and may have limited accessibility. Biofluid biomarkers do not provide anatomic information and report on the state of the patient at the time of sample collection. Blood and CSF collection are less expensive and more widely available [12]. Blood sampling is the most convenient and can be done multiple times during a trial. These considerations influence drug development and COU decision making. Imaging studies with PET, for example, will require an adequate number of trial sites with access to the PET ligand to be used. In clinical trials, use of combinations of imaging, CSF, and blood-based biomarkers is usually most informative of the biological impact of the intervention.

## Roadmap for Biomarkers in AD Drug Development

**Fig. 1:**
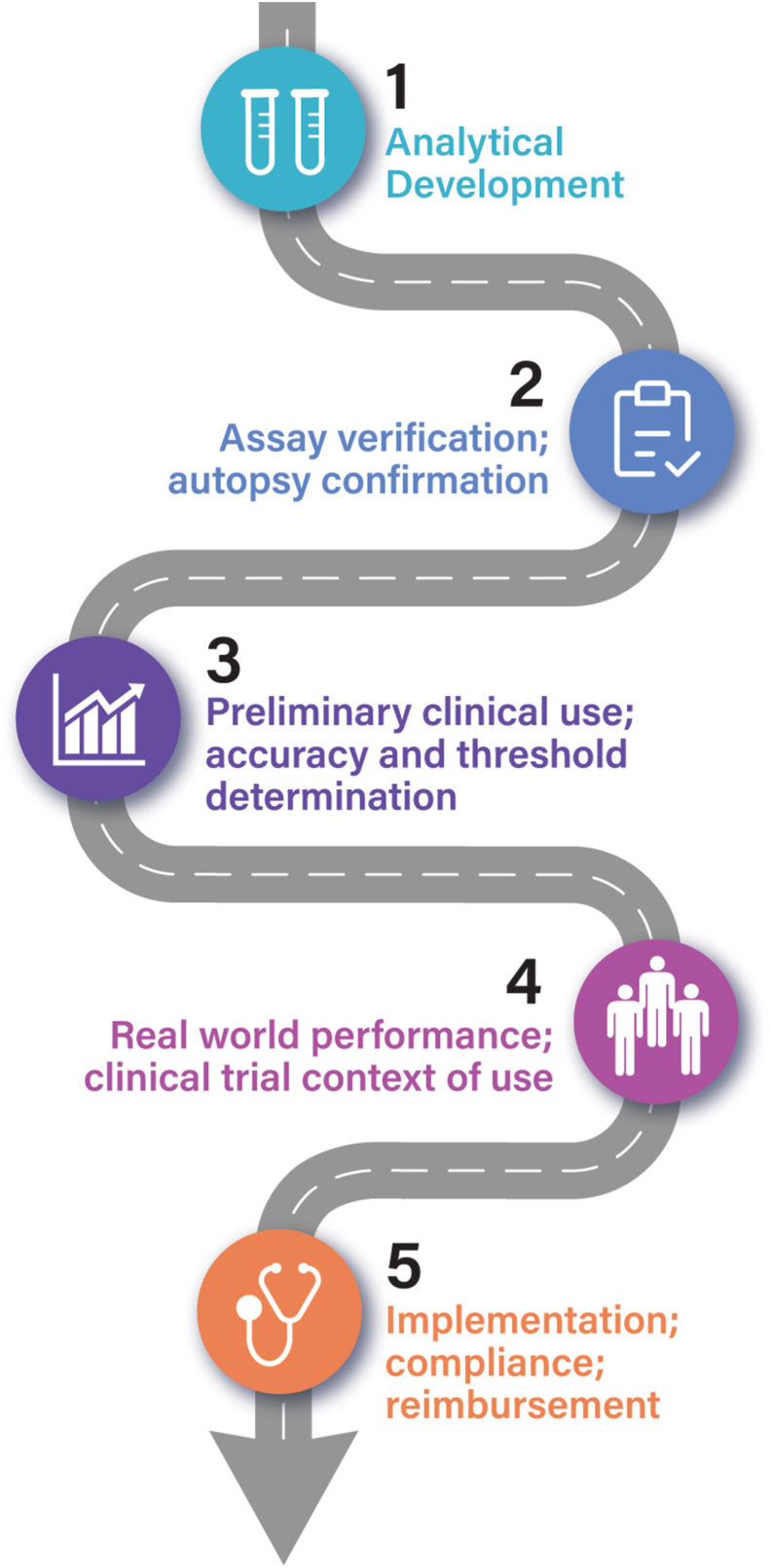
Road map for biomarker development from assay creation to implementation in clinical care and clinical trials (© J Cummings; M de la Flor, PhD, Illustrator).

Biomarker development is complicated, and there are many steps between assay development and clinical deployment (Fig. 1). Five major steps have been described in biomarker development [13]. The process begins with establishing the analytical validity of the proposed biomarker. The next step further defines the assays and establishes the biomarker performance characteristics. The third step advances understanding of accuracy in healthy controls and individuals with cognitive impairment and develops preliminary interpretive thresholds. Next, the real-world performance of the biomarker in terms of positive and negative predictive value, sensitivity and specificity, area under the curve, and accuracy are determined. This phase may include the preliminary introduction of the biomarker into longitudinal cohorts or clinical trials. The final phase of development establishes the biomarker's utility through widespread implementation and real-world use. This step is comprised of use in clinical trials, implementation in clinical care, study of compliance and adherence in real-world world settings, and cost and reimbursement considerations.

Biomarkers used in clinical trials may be in different stages of validation. The credibility of the data for each COU will depend on the quality and comprehensiveness of the available validity and accuracy information.

## Biomarkers Across the Phases of Drug Development Programs

COUs for biomarkers are used throughout all phases of drug development and will differ among programs depending on the mechanism of action of the test agent, population of interest, outcomes chosen, and goals of each trial. Diagnostic biomarkers may be needed for all phases. Target engagement biomarkers may be more useful in Phase 2, and disease impact biomarkers may be more informative in Phase 3. We present the biomarkers typically used in different phases acknowledging that the COU of biomarkers will vary among development programs.

## Biomarkers in Phase 1 Clinical Trials

Phase 1 clinical trials are first-in-human exposures of candidate therapies assessed previously in non-clinical studies. Phase 1 trials include single ascending dose (SAD) and multiple ascending dose (MAD) studies and biomarkers play important roles in these early-stage trials.

Phase 1 trials of small molecules are usually conducted with healthy volunteers. In some Phase 1 programs, a Phase 1b study including an older cohort or a cohort with AD will be included in the MAD studies once a target dose has been established in younger healthy individuals. Biomarkers contribute to establishing the safety profile of the candidate therapy. Liver function tests, electrocardiography, complete blood counts, and other laboratory assessments are the key biomarkers in healthy volunteers.

Phase 1 studies establish the pharmacokinetic profile of the candidate therapy (half-life, maximum concentration, time to maximum concentration, blood brain barrier penetration, etc) and sometimes include pharmacodynamic studies of target engagement. For example, Phase 1 studies of healthy volunteers helped define the effect of secretase inhibitors on amyloid in anti-amyloid development programs. Amyloid beta-protein (Aβ) production is continuous in healthy individuals, and production and clearance can be measured using stable isoleucine kinetics (SILK) in healthy volunteers or patients with AD. In this approach, CSF sampling follows administration of an isotope-labeled amino acid (^13^C_6_-leucine). High-resolution tandem mass spectrometry quantifies the labeled Aβ, revealing rates of Aβ synthesis and clearance [14]. Phase 1 studies of a gamma-secretase inhibitor (LY450139; semagecestat) in healthy volunteers established the pharmacokinetic and dose-exposure relationships for this agent [15]. Later studies failed to confirm the therapeutic benefit of this approach [16].

Phase 1b clinical trials of biological therapies such as MABs and tau-directed antisense oligonucleotides (ASOs) are conducted in individuals with symptomatic AD (not in healthy participants) and include demonstration of target engagement. If amyloid is a target of therapy as in the case of the amyloid plaque-lowering MABs, amyloid PET is used as an entry criterion as well as a pharmacodynamic response biomarker. The Phase 1b PRIME study of aducanumab provided the first evidence of marked amyloid plaque reduction by an anti-amyloid MAB [17]. Amyloid PET served as a diagnostic biomarker and pharmacodynamic response biomarker establishing a central role for this biomarker technology in anti-amyloid MAB drug development.

Tau biomarkers played a critical role in the Phase 1 MAD study of BII080, an intrathecally administered ASO directed at selectively reducing microtubule associated protein tau (MAPT) messenger RNA (mRNA) to decrease the synthesis of tau protein. Reductions in CSF total tau, CSF p-tau 181, and neurofibrillary tangles burden (tau PET) supported target engagement [18,19].

These early-stage Phase 1b studies provided key information to guide drug development decisions by informing the dose and timing of drug administration and choice of biomarker and clinical outcomes for Phase 2 studies.

## Biomarkers in Phase 2 Clinical Trials

### Diagnostic context of use

Participant diagnosis is a major COU for biomarkers in AD drug development. Approximately 50 % of patients with MCI have biologically confirmed AD and approximately 75 % of patients diagnoised cinically with mild AD dementia have confirmatory biomarkers consistent with an AD diagnosis [20]. Without diagnostic biomarkers, a substantial number of non-AD participants are entered into “AD” clinical trials. These participants may progress at different rates, respond differently to interventions, and have different safety and tolerability profiles than participants with confirmed AD. Inclusion of these patients in clinical trials could lead to erroneous conclusions concerning drug efficacy and safety. FDA guidance on clinical trials in early AD states that recent biologically based diagnostic criteria should be used to confirm the diagnosis of AD in DTT development programs [21].

Diagnostic confirmation of AD uses the amyloid, tau, neurodegeneration (AT(N)) framework. The framework includes core diagnostic elements of AD (e.g., amyloid and tau) as well as measures of pathophysiological changes that occur in AD but are not AD-specific (e.g., inflammation, neurodegeneration), and co-pathology such as alpha-synuclein and vascular pathology [22]. Diagnosis can be based on amyloid PET, CSF amyloid-beta protein (Aß) ratios (p-tau 217/Aß42, t-tau/Aß42, Aß42/40), or plasma p-tau 217 or p-tau 217/Aß42 ratios. Positive tau PET predicts the presence of positive amyloid PET in nearly all cases, and tau PET is sufficient for a diagnosis of AD in most circumstances [23]. Diagnosis using blood-based biomarkers may require additional testing to avoid over-reliance on a single blood test or when the plasma level of the biomarker is in an indeterminant zone between defined levels of positive and negative.

Ethnoracial factors affect diagnostic decisions based on biomarkers. In the US, cognitively normal non-Hispanic Black individuals at-risk for AD are less likely to have abnormal brain amyloid compared to non-Hispanic Whites. The presence of more African ancestry genes is associated with lower likelihood of having brain amyloid [24]. Given the lower rates of amyloid PET positivity, screen fail rates for trial participation using PET will be higher among non-Hispanic Blacks. The relationship between amyloid PET positivity and elevated plasma p-tau 217 is the same across these populations [25]. Screening with plasma p-tau 217 and collecting amyloid PET only on individuals with elevated levels will yield the same rate of positive amyloid PET as observed in White participants.

The use of amyloid and tau biomarkers to confirm the diagnosis of AD is independent of the mechanism of action of the candidate therapy. Drugs with non-amyloid non-tau mechanisms require diagnostic confirmation to assure the presence of AD pathology regardless of the mechanism of the test agent.

### Prognostic context of use

Assurance of decline in the placebo group is an important element of designing Phase 2 trials, and prognostic biomarkers provide information regarding the likelihood that an individual will evidence clinical decline. Elevated plasma levels of p-tau 217, p-tau 181, or glial fibrillary acidic protein (GFAP) predict a more rapid decline from MCI to AD dementia; neurofilament light (NfL) levels or Aß 42/40 ratios do not provide additional prognostic insight [26,27]. Of these biomarkers, p-tau 217 consistently correlates better with CSF biomarkers and is better aligned with cognitive decline [28].

Positive tau PET predicts future cognitive decline in amyloid positive asymptomatic individuals, and the combination of tau PET and p-tau 217 strengthens the relationship between the biomarkers and onset of cognitive symptoms in asymptomatic individuals [29,30]. Tau PET predicts decline to AD dementia among those with MCI due to AD [31].

Enriching an AD clinical trial population by requiring biomarker evidence of likely fast progression can reduce the sample size required to observe a drug-placebo difference by 25–40 % [32,33].

### Risk/susceptibility context of use

The major biomarkers for the risk COU in AD clinical trials involve identifying individuals with specific risk alleles or autosomal dominant mutations. Carriers of the apolipoprotein E ε 4 (*APOE4*) gene are more likely to develop AD and to do so at a relatively younger age than *APOE4* non-carriers. Homozygotes typically have an earlier onset than heterozygotes. Drugs designed to intervene in the adverse effects of the APOE4 protein will use this genotype or corresponding proteotype to identify appropriate participants [34].

Presenilin 1 (PS1), presenilin 2 (PS2), and amyloid precursor protein (APP) mutations are trait biomarkers of autosomal dominant AD [35]. They are present from conception and are fully penetrant with few exceptions. Mutation carriers typically develop symptomatic AD at an earlier age than those with the non-genetic form of the disease. Asymptomatic and symptomatic mutation carriers can be studied to assess the prevention capability of interventions in the asymptomatic group (e.g., delay to symptom onset) and treatment capabilities in the symptomatic group (e.g., slowing of cognitive decline) [36]. Biomarker changes in individuals with autosomal dominant AD begin in midlife and follow a course like that of late onset AD. Use of biomarkers in trials of those with hereditary AD adhere to the COU applications of trials in the last-onset form. Ethical issues can arise when considering patient selection based on genetic or biomarker information. If required for scientific validity, blinding of trials can be accomplished by including people with and without the genetic abnormality and assigning those without the abnormality to receive placebo [37]. Alternatively, evidence suggests that biomarker disclosure to potential trial participants may be feasible without affecting trial integrity [38,39].

Metabolic factors such as diabetes, obesity, hypertension, hypercholesterolemia, and stroke increase the risk of AD [40]. Few Trials have used these cardiovascular and metabolic risk factors to define trial populations.

### Predictive context of use

Predictive biomarkers forecast the response to treatment --- benefit or harm. There are few predictive biomarkers for clinical trials of AD. The presence of the *APOE4* genotype is associated with an increased risk of amyloid-related imaging abnormalities – edema type (ARIA-E) and ARIA-hemorrhagic (ARIA-H) type among patients receiving anti-amyloid MABs, and the risk is greatest in those with two copies of the gene. *APOE4* genotype or proteotype is a predictive biomarker of potential harm associated with anti-amyloid MAB therapy [[41], [42], [43]].

In the Phase 2 Trailblazer-ALZ study, it was hypothesized that patients with high tau levels (standard uptake value ratio (SUVR) greater than 1.46) might not respond to treatment with donanemab. Patients with high tau levels were excluded from the trial [44]. The Phase 3 Trailblazer-ALZ 2 trial included those with high tau levels and observations supported the hypothesis that patients with high level of tau as measured by flortaucipir PET at baseline responded less well to donanemab than patients with low to medium levels of tau at baseline [45]. These studies suggest that tau PET may serve as a predictive biomarker identifying patients who are less likely to respond or have less robust responses than patients with lower tau levels.

Experimental attempts to use mechanism-related biomarkers to predict clinical benefit are being pursued. For example, a Phase 1b/2a trial interrogating the anti-inflammatory effects of autologous mesenchymal stem cells requires elevated inflammation biomarkers --- C-reactive protein (CRP), interleukin (IL)-6, tumor necrosis factor (TNF)-alpha, or erythrocyte sedimental rate --- for trial participation (NCT06775964).

Predictive biomarkers differ from prognostic biomarkers. The latter are biomarkers that allow inferences regarding disease course (e.g., progression to MCI predicted by p-tau 271 [29]. Predictive biomarkers are indicators of a therapeutic response if a specific treatment is instituted [4]. There are a substantial number of prognostic biomarkers for AD and relatively few predictive biomarkers.

### Pharmacodynamic context of use

The pharmacodynamic COU is a key use of biomarkers AD trials, allowing the assessment of efficacy of the test agent. Table 2 summarizes the principal pharmacodynamic biomarkers used in AD clinical trials [46]. Most biomarkers in Table 2 have been widely used in clinical trials and are described in this review; some biomarkers have been used in relatively few trials or only in preliminary studies and should be regarded as emerging measures. If biomarkers are the primary outcomes of the Phase 2 trial, they may inform the go/no go decision as to whether to stop an agent, collect additional information, or advance to Phase 3. Two types of pharmacodynamic data are collected in clinical trials. Target engagement biomarkers determine if there is a direct physiologic interaction of the target with the test agent; disease impact biomarkers reveal if the intervention affects the biological features of the disease. For anti-amyloid MABs, the target engagement biomarker (amyloid PET) is the same as the disease impact biomarker since amyloid plaque is both the treatment target and one of the defining pathophysiological features of AD [47].

**Table 2:** Pharmacodynamic biomarkers used in Alzheimer's disease (AD) clinical trials (established and emerging biomarkers of possible use in future trials are included in the table; not all biomarkers are equally well validated).

Biomarker Class	Pharmacodynamic Biomarkers Implemented in AD Trials
Amyloid	• Amyloid PET • CSF: p-tau 217; p-tau 181/Aß 42; total tau/Aß 42; Aß 42/40 • Plasma: p-tau 217
Tau	• Tau PET • Plasma total tau, p-tau 181, p-tau 217, brain-derived tau, MTBRtau-243 • CSF p-tau 181, p-tau 217, pre-tangle p-tau 262, p-tau 356, CSF tau368/t-tau, MTBRtau-243
Neurodegeneration	• MRI • Plasma NfL, total tau • CSF NfL total tau, neurogranin, visinin-like-protein 1
Inflammation	• TSPO PET • Plasma GFAP, TNF-alpha, complement factor 3 and complement factor 4 • CSF GFAP, YKL-40, sTREM2, TNF-alpha, complement factor 3 and complement factor 4, monocyte chemoattractant protein 1/chemokine ligand 2 ratio; interferon gamma–inducible protein-10/motif chemokine ligand 10 ratio, IL-1, IL-6, IL 8, IL-18, IL-1ß, secreted modular calcium-binding protein 1, osteopontin
Synaptic integrity	• Synaptic vesicle protein 2A (SV2A) PET • Fluorodeoxyglucose PET • Electroencephalography • Functional MRI • Plasma neuroregulin 1 • CSF SNAP-25,synaptotagmin-1, growth-associated protein-43, RAB3A, synaptophysin, synaptopodin, neurogranin, vesicle-associated membrane protein 2, neuronal pentraxin, postsynaptic density protein 95
Vascular and blood brain barrier integrity	• MRI • Plasma vascular cell adhesion molecule-1, intercellular adhesion molecule-1, placental growth factor • CSF vascular cell adhesion molecule-1, intercellular adhesion molecule-1, placental growth factor, soluble platelet-derived growth factor receptor ß

GFAP - glial fibrillary acidic protein (GFAP); IL - interleukins; MRI – magnetic resonance imaging; MTBRtau-243 - Microtubule Binding Region (MTBR)tau-243; NfL - Neurofilament light chain; PET – positron emission tomography; SNAP-25 - synaptosome-associated protein 25; TNF-alpha - tumor necrosis factor-alpha; TREM2 - soluble triggering receptor expressed on myeloid cells 2; TSPO – translocator protein.

A surrogate biomarker is a pharmacodynamic measure whose change is associated with a clinical response with such high consistency that assessments of clinical outcomes are obviated. There are no fully validated surrogate biomarkers for AD clinical trials. Reduction in Aß plaques as measured by amyloid PET is considered “reasonably likely” to predict clinical benefit based on data derived from several anti-amyloid MAB clinical trials involving participants with early AD, and plaque clearance was used as the basis for accelerated approval of aducanumab and lecanemab [48]. Lecanameb later obtained standard approval following demonstration of clinical benefit in a Phase 3 trial [49]. To consider accelerated approval of a new therapy, FDA requires demonstration of the biological plausibility of the relationship among the disease, end point, and desired effect. The evidence may include epidemiologic, pathophysiologic, therapeutic, pharmacologic, or information from biomarkers or other relevant tools [50].

Non-amyloid biomarkers may provide information comparable to the data that led to use of amyloid PET as reasonably likely to predict clinical benefit. This step will require the convergence of observations from multiple clinical trials. Tau PET provides diagnostic and prognostic information and correlates with cognitive decline [51]. If multiple therapeutic trials establish a reproducible relationship between reduction of tracer signal on tau PET and clinical benefit, requirements may be met for use of tau PET as a surrogate marker reasonably likely to be associated with clinincal benefit. Fluid biomarkers that have a plausible relationship to the biology of AD, are consistently related to clinical outcomes, and have supportive data across multiple trials and drug mechanisms could obtain surrogate status.

### Monitoring context of use

Monitoring biomarkers are collected serially during a trial to document the longitudinal effects of the intervention on the disease process as reflected in changes in plasma, CSF, or imaging measures [7]. There have been relatively few trials of sufficient duration (e.g., 18–24 months) with serial collection of biomarkers to inform best practices for monitoring. In the Phase 2 trial of lecanemab, reductions were observed in p-tau181 at months 12 and 18 compared to an absence of change from baseline in the placebo group [52]. Plasma p-tau 217 was used to monitor the treatment effects in the Phase 2 trial of donanemab. Samples were collected at weeks 12, 24, 36, 52, 64, and 76. Drug-placebo differences were evident by week 12. At week 76, there was a 23 % decrease in p-tau 217 levels in those receiving donanemab compared to a 6 % increase in p-tau 217 levels in those on placebo [53]. A similar pattern was observed with serial monitoring of GFAP in the Phase 2 donanemab trial. There was a 12 % decrease in GFAP levels in the active therapy group and a 15 % increase in the placebo group during the 76-week trial [53]. Use of p-tau 217 to monitor the response to treatment in the 18 month Phase 3 trial of donanemab confirmed the Phase 2 observations [45]. These studies provide preliminary evidence that serial monitoring with plasma biomarkers can document sequential drug-placebo differences during an AD trial.

### Safety context of use

Magnetic resonance imaging (MRI) is used to detect ARIA that may occur during treatment with anti-amyloid MABs. Serial MRIs are collected on a schedule determined for each MAB. ARIA occurrence is highest during the introductory phase of the MAB treatment, and MRI monitoring is most frequent during this time [54,55].

Biomarkers such as liver function tests, electrocardiographic abnormalities, and tests for adverse marrow and renal effects of drugs comprise core elements required for safe drug development in clinical trials.

## Biomarkers in Phase 3 Clinical Trials

The emphasis of biomarkers may shift between Phase 2 and Phase 3. With few exceptions, such as accelerated approval, the key outcome of Phase 3 trials is a drug-placebo difference on the prespecified primary clinical outcome. Diagnostic biomarkers confirm the presence of AD and pharmacodynamic biomarkers in Phase 3 provide supportive evidence of efficacy and comprise the basis for demonstrating the biological impact of the intervention on the disease. Pharmacodynamic biomarkers of the disease impact type are more important than mechanistic target engagement biomarkers in Phase 3 (Table 2).

Prognostic biomarkers may be used to enrich Phase 2 populations and to help ensure sufficient decline in the placebo group and facilitate demonstrating a drug-placebo difference. Prognostic biomarkers may not be employed in Phase 3 since they may narrow the product labeling to use only in those with a positive prognostic marker [4].

## Pharmacodynamic Biomarkers in AD Trials

Amyloid PET demonstration of Aß plaque reduction is the primary pharmacodynamic biomarker for anti-amyloid MABs. In trials of the anti-amyloid MABs lecanemab and donanemab, approximately 80 % or more of participants receiving active treatment had treatment-related amyloid clearance (TRAC) with negative amyloid PET scans by visual read at trial end [56]. Gantenerumab, an anti-amyloid MAB that did not achieve its primary endpoint, had TRAC in 28 % and 26.8 % of participants in the Phase 3 Graduate I and II trials [57]. Clearance to a negative visual read (or Centiloid level of 25 or less) corresponds to slowed clinal decline [58].

An increase in CSF and plasma Aß 42/40 is anticipated with plaque dissolution, reduced plaque formation, and increased levels of the soluble forms of Aß [59]. This was observed in trials with lecanemab, gantenerumab, and aducanumab [49,56,60,61]. A trial-ready biomarker for Aß oligomers --- considered to be the most toxic species of Aß --- is not available [62].

Small molecule approaches to amyloid targeting in AD include inhibition of aggregation with plasma p-tau, Aß42, or AB 42/40 as key outcome biomarkers [63]. Similarly, trials of beta secretase and gamma secretase inhibitors are expected to demonstrate decreased Aß production as shown by plasma Aß42 or Aß 42/40 (target engagement biomarkers) [16,64].

**Fig. 2:**
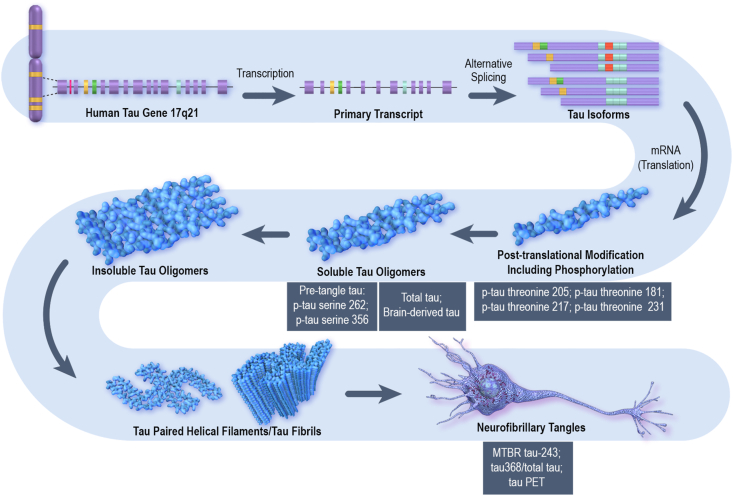
Continuum of forms of tau pathology in the Alzheimer's disease brain from the microtubule associated protein tau (MAPT) gene to the insoluble neurofibrillary tangle and the corresponding biomarkers that may be used in clinical trials (© J Cummings; M de la Flor, PhD, Illustrator).

There is an increasing repertoire of pharmacodynamic tau-related biomarkers applicable to clinical trials of potential tau-directed therapeutics (Fig. 2; Table 2). Tau protein undergoes multiple types of post-translational modification including phosphorylation and several types of phospho-tau with phosphorylation at different tau protein epitopes are detectable in CSF and plasma. Current studies indicate that phosphorylation of tau at threonine 231 (p-tau 231) increases early in the AD continuum, followed by increases later in phosphorylation at threonine 217 (p-tau 217), and then at threonine 181 (p-tau 181), followed by phosphorylation at threonine 205 (p-tau 205) [65]. These initial increases in p-tau occur approximately in concert with an elevated Aß plaque burden observed with amyloid PET and before the formation of significant neurofibrillary burden associated with positive tau PET. CSF pre-tangle p-tau with phosphorylation at serine residues 262 and 356 report on later stages of the tau protein aggregation continuum [66]. Microtubule Binding Region tau 243 (MTBRtau-243) and tau PET reflect the presence of neurofibrillary tangles in the brain [67,68]. Similarly, CSF tau368/t-tau correlates highly with tau PET and constitutes another fluid measure reflective of tangle pathology [69]. Measures of brain derived tau distinguish between tau of peripheral and central origin [70]. Phospho-tau 217 is thought to originate from peri-plaque neurons subject to amyloid-related tau dysregulation and shows a high correlation with both amyloid PET and tau PET [71,72].

Trials of the microtubule-associated protein tau (MAPT) ASO MAPT_Rx_ (BIIB080) used tau biomarkers to show reduced total tau and reduced p-tau 181 in CSF and plasma and reduction of the neurofibrillary tangle burden on tau PET in response to treatment [19].

Markers of neurodegeneration are often collected in AD clinical trials as pharmacodynamic biomarkers. They are not specific to AD and occur across neurodegenerative disorders (NDDs) [73]. NfL reflects axonal injury, and total tau and visinin-like protein 1 (VILIP-1) are measures of neuronal injury and death [[74], [75], [76]]. MRI measures of total brain volume, hippocampal volume, ventricular volume, or cortical thickness in AD assess brain atrophy and often reflect neuronal loss although there are other causes of reduced brain volume [77].

Inflammation is present in the brains of patients with AD and other NDDs, and reduced inflammation is an important pharmacodynamic biomarker in trials of putative anti-inflammatory agents. The mechanism of action of the agents in trials include antibody agonists of targeting triggering receptor expressed in myeloid cells (TREM2), microglial modulators, and agents aimed at reducing levels of toxic cytokines induced by microglial and astroglial activation [78,79]. CSF measures of chitinase-3-like protein 1 (YKL-40) and CSF and plasma measures of GFAP are astroglial activation markers commonly elevated in inflammatory states [80]. Trials of semaglutide, a glucagon-like protein 1 (GLP-1) agonist with anti-inflammatory properties, are collecting a broad array of inflammatory markers to allow construction of a comprehensive anti-inflammatory response profile for this agent (Table 2). Plasma biomarkers to be collected include GFAP, sTREM2, C-reactive protein (CRP), and NfL [81]. The current trials may provide insight into the most informative inflammatory biomarkers for clinical trials of agents targeting this process.

Translocator protein (TSPO) PET is considered a measure of activated microglia and is being investigated as an imaging biomarker for AD and other NDD possibly applicable in clinical trials. Insufficient affinity of the ligand for TSPO, and the low level of blood brain barrier penetration have limited the robustness of this biomarker. TSPO PET is being used as an outcome of a clinical trial for a colony stimulating factor 1 receptor inhibitor and microglial modulator (NCT06745583) and to monitor anti-inflammatory effects of autologous mesenchymal stem cells (NCT06775964).

Pharmacodynamic biomarkers are embedded in trials of treatments targeting synaptic plasticity in AD (Table 2). Presynaptic biomarkers include synaptosome-associated protein 25 (SNAP-25), synaptotagmin-1, growth-associated protein-43 (GAP-43), RAB3A, vesicle-associated membrane protein 2 (VAMP-2; synaptobrevin-2), neuropentraxin 2 (NPTX2), neuroregulin 1 (NRG1), and synaptophysin. Neurogranin and postsynaptic density protein 95 (PSD-95/DLG4) are postsynaptic biomarkers [[82], [83], [84], [85], [86], [87], [88]]. Although trial experience with synaptic markers is limited, CSF neurogranin levels decreased compared to placebo over 116 weeks of treatment with the anti-amyloid MAB, gantenerumab. CT1812 is a sigma-2 receptor antagonist that lowers the affinity of Aß oligomers for synaptic receptors [89]. Synaptic biomarkers neurogranin and synaptotagmin-1 decreased in participants receiving active treatment for 28 days compared to those on placebo. LM11A-31 is a p75 modulator targeting degeneration signaling. In a 26-week trial, LM11A-31 ameliorated the increase in SNAP-25 and neurogranin observed in the placebo group [90]. Data from additional trials are needed to inform the profile and magnitude of effects expected to predict a clinical benefit.

Synaptic vesicle protein 2A (SV2A) imaging reveals deceased synaptic abundance in AD and is a promising clinical trial tool [91]. SV2A PET has been used in exploratory trials of agents targeting synaptic plasticity [92]. The fluorodeoxyglucose (FDG) PET signal is derived from neuronal and synaptic activity and can be used as an indirect measure of synaptic function in clinical trials of AD [93]. Synaptic function participates in the cerebral circuitry responsible for the generation of electroencephalographic (EEG) rhythms. Analyses of EEG could contribute to assessing impairment and restoration of synaptic plasticity [94].

Pharmacodynamic biomarkers of vascular pathology are emerging. Vascular changes in AD include deposition of amyloid in the cerebrovascular wall and compromise of the blood brain barrier [95]. Relatively few biomarkers for vascular or blood brain barrier integrity are available and trial ready. Among those collected in some trials include MRI evidence of ischemic injury, vascular cell adhesion molecule-1 (VCAM-1), intercellular adhesion molecule-1 (ICAM-1), placental growth factor, and platelet-derived growth factor receptor ß (sPDGFRβ)(Table 2). The CSF/serum albumin ratio may provide information regarding blood brain barrier function [96].

## Biomarker Data Analytic and Interpretation Strategies

The statistical analysis plan (SAP) for biomarkers must be as carefully detailed as that for clinical outcomes (Table 3). A plausible pathophysiological model of AD guides both the biomarker choices and interpretation strategies. Biomarker results are reviewed for the magnitude of the drug-placebo difference, directionality and consistency of responses, dose-response relationships, and relationships to clinical outcomes. Group level correlations between clinical and biomarker endpoints examine the drug-placebo difference for biomarker and clinical endpoints. At the individual subject level, correlations assess relationships between change from baseline in biomarkers versus change from baseline in clinical endpoints [97]. These analyses apply to global, cognitive, and functional endpoints and their relationships to key biomarkers.

**Table 3:** Analysis and interpretation of biomarker outcomes in clinical trials of Alzheimer's disease (AD).

• Biomarker observations ○ Magnitude of the drug-placebo difference for biomarkers for the treatment related context of use ○ Relationship and consistency of the biomarker measures to the clinical outcomes (specified below) ○ Dose-response relationships of the magnitude of the drug-placebo difference ○ Directionality and consistency of the biomarker response across multiple biomarkers ○ Confirmation of the ability of predictive biomarkers (if any) to predict the response to the test agent ○ Relationship of the treatment related biomarker outcomes to the prognostic biomarkers if a range of prognostic biomarkers was present among participants at baseline • Analyses of the biomarkers collected in the trial ○ Pre-specification of the biomarker statistical analysis plan ○ Correlations among biomarkers ⁃ Order and strength of relationships among biomarker outcomes ○ Clinical relationships ⁃ Group level correlations are assessed using the drug-placebo difference for biomarkers versus drug-placebo difference on clinical endpoints (interpretation is dependent on the biomarker and disease stage) ⁃ Subject level correlations use change from baseline in biomarkers versus change from baseline in clinical endpoints (interpretation is dependent on the biomarker and disease stage) • Comparison of trial biomarker outcomes and outcomes reported from other trials ○ Examination of biomarker outcomes to those reported in trials of agents with similar mechanisms of action (magnitude, profile of changes, relationships/correlations among biomarker changes) ○ Examination of relationship of biomarker outcomes to those reported in trials of agents with alternative mechanisms of action (magnitude, profile of changes, relationships/correlations among biomarker changes) ○ If other agents have had regulatory review, consider the applicability of the review to biomarker results of the current trial

Biomarkers included in clinical trials may be primary, secondary, or exploratory outcomes. Biomarkers with limited validation data are typically included as exploratory markers. Information generated on exploratory markers can be used to justify their inclusion for COUs in future trials.

An important caveat in considering biomarker outcomes is that in most cases a specific magnitude of drug-placebo difference or specific threshold required for predicting clinical change has not been defined. Statistically significant changes are not necessarily associated with clinical benefit. The biomarker change is interpreted in terms of changes observed in other biomarkers and in clinical assessments.

Once biomarkers within a trial are thoroughly understood they can be compared to biomarker outcomes in trials with agents of similar mechanisms of action. For example, the threshold for amyloid reduction observed on amyloid PET was established by interrogating the results of outcomes from several trials of anti-amyloid MABs [58].

Comparison of biomarker outcomes from trials of agents with different mechanisms of action can be informative, but caution is required as alternative mechanisms of action might have different biomarker effects making comparisons of uncertain value. Input from regulatory reviewers on relevant biomarker outcomes --- within the program and across programs --- guide AD drug development decision making.

## Emerging Use Cases for Biomarkers in AD Trials

### Biomarkers in clinical trials for combination therapies

Approved DTTs slow AD progression by approximately 30 %. Optimizing patient function at the highest level for the longest time will require greater therapeutic efficacy. This may be achieved with highly efficacious monotherapies or combination therapies comprised of two or more novel agents or novel agents added to an anti-amyloid MAB [98,99]. A variety of design strategies can be considered for combination therapy development including 2 × 2 factorial designs assessing each agent and the combination compared to placebo, noninferiority designs comparing a novel agent to standard of care (including anti-amyloid MABs), or 3 arm designs comparing a combination of two novel agents to each component-agent of the combination [100]. The appropriate design will depend on the goals of the trial and the stage of the program.

Anti-amyloid MABs produce marked changes in biomarkers including reduction of Aß plaque burden and decreased plasma p-tau 181, p-tau 217, GFAP, and CSF neurogranin. These substantial changes make it challenging to detect any additional biomarker shift produced by a novel agent given in conjunction with an anti-amyloid MAB. Target engagement biomarkers specific to the mechanism of action of the novel agent may assist in demonstrating a unique pharmacologic aspect of the add-on intervention. Measurement of biomarker changes in early-stage (Phase 1b/2a) trials prior to utilization in conjunction with an anti-amyloid MAB may allow demonstration of the biomarker profile of the novel intervention. Analytic strategies may help distinguish between biomarker changes produced by anti-amyloid MABs and biomarker changes in add-on combinations such as the magnitude, profile, onset and trajectory, and the durability of biomarker effects.

### Biomarkers in platform trials

Platform trials are a highly efficient mechanism for advancing AD drug development [101]. A closely monitored, constantly improving site network is engaged in continuous clinical trials of multiple agents that matriculate to later stage trials, are entered into combinations, or are stopped from further development [102]. Pooled data from a shared placebo group minimize the number of participants assigned to placebo. Bayesian adaptive features may be built into the trial platform to further optimize drug development efficiency [103].

Biomarkers can be integrated into platform trials [104]. Biomarker-driven adaptive trials identify subpopulations based on biomarkers and evaluate the effectiveness of the treatment on that subpopulation in a pre-specified statistically valid manner. The pattern and magnitude of biomarker changes can be assessed across multiple types of interventions as well as across biomarker-defined subgroups. New biomarkers can be introduced into the platform and comparative studies of biomarkers can be conducted [105].

There is relatively limited experience with platform trials in AD. Key challenges to be resolved in platform trials include how to manage blinding if agents with different formulations are to be tested (e.g., intrathecal administration, intravenous administration, etc) and how to ensure that patients on placebo for part of the platform exposure have the opportunity for active therapy as new agents are integrated into the platform.

## Emerging Candidate Biomarkers for Alzheimer's Disease Clinical Trials

Several types of biomarkers are not yet playing key roles in AD clinical trials but are expected to be increasingly influential in AD drug development. Proteomics, measurement of exosome biomarkers, EEG, ocular markers, and digital biomarkers are among the areas where use in AD clinical trials is anticipated. Some of these approaches have had extensive testing in longitudinal cohorts, non-interventional clinical studies, and small trials but have not yet had extensive use in large interventional trials.

Proteomics-defined subtypes of AD have been identified and may represent a means of identifying subpopulations with specific treatment response profiles [106]. Drug induced changes in proteomic profiles are evident in preliminary studies and may comprise a means of exploring treatment response characteristics [107]. Artificial intelligence is increasingly used in interrogation of proteomic data to define their role in diagnosis, risk state identification, treatment effect, and longitudinal monitoring [108,109]. Proteomics have been used in preliminary studies to identify proteomic response profiles of therapeutic interventions [107,110,111].

Exosomes --- nano-sized extracellular vesicles created and secreted by cells through the fusion of multivesicular bodies with the plasma membrane --- play a critical physiological role by transporting nucleic acids, proteins, lipids, metabolites, and other essential biomolecules to both local and distant organelles and cells [112]. Neuron-derived exosomes from patients with AD contain pathology-related protein cargo consisting of tau, p-tau, Aβ, TDP-43, inflammatory proteins, or α-synuclein. If analytical development supports their wide deployment as biomarkers, exosomes may allow detection of co-pathology common in AD and could become key in trial planning and participant selection.

EEG utilizes differential amplifiers to record voltage of oscillatory electrical fields generated by cortical neurons. Recent advances in EEG technology make this technology more viable as a trial biomarker. Computerized analysis for Quantitative EEG (QEEG), provides power spectra and mapping of the wavelength frequency bands (delta 0–3 Hz, theta 4–7 Hz, alpha 1 8–10 Hz, alpha 2 10–13, beta 13–30, gamma 30–70) that can be mapped onto a brain template to give spatial as well as frequency information [113]. Measures of absolute and relative power as well as metrics of connectivity provide additional information relevant to synaptic, neuronal, and circuit function [114]. Preliminary studies demonstrate QEEG differences between predementia patients with and without brain amyloid suggesting a role for these measures in pharmacodynamic and monitoring COUs [115]. Agents targeting synaptic function have used EEG to assess circuit function as a measure of efficacy [116].

AD is often accompanied by changes in the eye, giving rise to the opportunity to develop ocular and retinal biomarkers with potential roles in early detection and diagnosis. Optical coherence tomography (OCT) measures of retinal fiber layer thickness, OCT angiography, saccadic eye movement prosaccade latency, and anti-saccadic errors are among the ocular assessments currently being pursued [117]. Incorporation in clinical trials as a means of documenting drug-placebo differences in pharmacodynamic response to therapy is a plausible COU.

There has been a marked growth in digital biomarker availability, but few have been integrated into clinical trials. Passive monitoring by smartphone may represent a sensitive means of detecting early cognitive impairment and triggering more definitive diagnostic assessments [118]. Actigraphy may be a monitoring biomarker to assess activity levels of patients on active treatment or placebo, and monitoring of vital signs may serve as a safety COU. Artificial intelligence and machine learning are required to manage and interpret the large amount of data produced by digital biomarkers, and incorporating digital biomarkers in trials will require new bioinformatic strategies [119].

## Summary and Conclusions

Anti-amyloid MABs have opened a new era of treatment of AD with DTTs [120]. Therapies addressing other targets either as monotherapies or used in combination with anti-amyloid MABs are under investigation. In concert with the evolution of new therapeutics are rapid advances in biomarkers including imaging, CSF assays, and blood-based measures that can accelerate clinical trials and the development of new therapeutics for AD. Biomarkers have varying COUs in Phase 1, Phase 2, and Phase 3 clinical trials. Pharmacodynamic biomarkers are essential to validate efficacy of target engagement or impact on fundamental disease processes. Prespecification of expected biomarker changes and biomarker data analytic strategies are critical to credible interpretation of biomarker findings. Emerging proteomic, exosomal, EEG, ocular, and digital biomarkers combined with artificial intelligence/machine learning computational approaches promise to further transform the AD clinical trial landscape providing information that will lead to faster drug development and more efficacious treatments for patients.

## Author contributions

• Conceptualization – JLC.

• Data generation – JLC, GA, ALO, AO.

• Investigation – JLC, SS, GA. ALO, AO.

• Supervision – JLC.

• Writing – original draft – JLC, SS, GA. AO.

• Writing – review & editing – JLC, SS, GA. AO.

• Approval of final version - JLC, SS, GA. ALO, AO.

## Declaration of competing interest

The authors declare the following financial interests/personal relationships which may be considered as potential competing interests:Jeffrey Cummings reports consultation to Acadia, Acumen, ALZpath, Annovis, Artery, Axsome, Biogen, Biohaven, Bristol-Myers Squib, Eisai, Fosun, GAP Foundation, Hummingbird Diagnostics. IGC, Janssen, Julius Clinical, Kinoxis, Lighthouse, Lilly, Lundbeck, LSP/eqt, Merck, MoCA Cognition, Novo Nordisk, NSC Therapeutics, Optoceutics, Otsuka, Praxis, ReMYND, Roche, Scottish Brain Sciences, Signant Health, Simcere, sinaptica, T-Neuro, TrueBinding, and Vaxxinity pharmaceutical, assessment, and investment companies. Jeffrey Cummings reports a relationship with National Institute of Neurologic Disorders and Stroke that includes: funding grants. Jeffrey Cummings reports a relationship with Alzheimer's Drug Discovery Foundation that includes: funding grants. Jeffrey Cummings reports a relationship with Ted and Maria Quirk Endowment that includes: funding grants. Jeffrey Cummings reports a relationship with Joy Chambers-Grundy Endowment that includes: funding grants. Jeffrey Cummings owns the copyright of the Neuropsychiatric Inventory with royalties paid to Jeffrey Cummings. Jeffrey Cummings is co-founder of CNS Innovations and Mangrove Therapeutics. Jeffrey Cummings is a member of the editorial boards of the Journal of Prevention of Alzheimer's Disease and Translational Neurodegeneration. Other authors declare that they have no known competing financial interests or personal relationships that could have appeared to influence the work reported in this paper.
